# The G2 erosion model: An algorithm for month-time step assessments

**DOI:** 10.1016/j.envres.2017.11.010

**Published:** 2018-02

**Authors:** Christos G. Karydas, Panos Panagos

**Affiliations:** a Senior Researcher in Geomatics, Mesimeri P.O. Box 413, 57500 Epanomi, Greece; b European Commission, Joint Research Centre, Directorate for Sustainable Resources, Via E. Fermi 2749, I-21027 Ispra, VA, Italy

**Keywords:** Soil loss, Sediment yield, Month-time step, Vegetation retention, Landscape alterations

## Abstract

A detailed description of the G2 erosion model is presented, in order to support potential users. G2 is a complete, quantitative algorithm for mapping soil loss and sediment yield rates on month-time intervals. G2 has been designed to run in a GIS environment, taking input from geodatabases available by European or other international institutions. G2 adopts fundamental equations from the Revised Universal Soil Loss Equation (RUSLE) and the Erosion Potential Method (EPM), especially for rainfall erosivity, soil erodibility, and sediment delivery ratio. However, it has developed its own equations and matrices for the vegetation cover and management factor and the effect of landscape alterations on erosion. Provision of month-time step assessments is expected to improve understanding of erosion processes, especially in relation to land uses and climate change. In parallel, G2 has full potential to decision-making support with standardised maps on a regular basis. Geospatial layers of rainfall erosivity, soil erodibility, and terrain influence, recently developed by the Joint Research Centre (JRC) on a European or global scale, will further facilitate applications of G2.

## Highlights

• G2 is a soil erosion model for developing monthly erosion maps at regional scale.
• G2 enhances the spatio-temporal variability of cover management and erosivity factors.
• The V-factor models the impact of vegetation cover, land use and imperviousness layer.
• G2 uses data from ESDAC, LUCAS, COPERNICUS, CORINE, SENTINEL and EU-DEM databases.
• G2 has been applied in Strymonas Catchment, Crete, Cyprus and Albania catchments.

## 1 Introduction

Erosion modelling is used in order to achieve a better understanding of erosion processes, provided that experimental conditions from which directly measured outcomes could be derived, are either impossible or impractical to create (Tolk, 2015). The importance and achievements of erosion modelling (either for soil loss, sediment yield, or both) have been argued by a plethora of research works; see the review of Merritt et al. (2003).

The wide spreading of geographic information systems (GIS) and use of remote sensing data has accelerated erosion model development significantly, as it allows for data input from multiple sources, easy model structure modifications, and unconditioned model rescaling (Giordano et al., 1991, De Vente and Poesen, 2005). According to Karydas et al. (2014), more than 80 erosion models have been developed for different purposes in half a century. Despite the wealth of erosion models and applications, though, selection of an appropriate model for operational mapping remains a difficult undertaking.

With a view to support regular monitoring by decision-makers involved in environmental and agricultural policies, the geoland2 project has developed the G2 erosion model, in the framework of the Copernicus Land Monitoring Service (http://land.copernicus.eu/) (former GMES). The development of a new erosion model was justified by the requirements for operational, standardised mapping solutions, raised by the new environmental policies in Europe, such as the Soil Thematic Strategy (Montanarella, 2015) and the Common Agricultural Policy (Panagos et al., 2016), in the view of rapid land use changes and the climate change effects. Fundamental in a new modelling approach would be a seasonal time-step (rather than averaged annual assessments), which could be accomplished by using regularly updated space-born data. On the other hand, a new model had to take advantage of previous experience, taking account of the urgent character of the monitoring tasks ahead and the potential high cost of creating new experimental data.

As a result, G2 was based on RUSLE and EPM heritage (for soil loss and sediment yield assessments, respectively), trying at the same time to overcome reported drawbacks of both models; for example, the questionable applicability in different areas from those where these models were developed or on a different temporal scale than annual, limitations to sheet and interill processes, etc. (see Kale and Vadsola, 2012). Considering that G2 adopts fundamental empirical equations from RUSLE and EPM, it can be classified as an empirical model, too.

G2 has been made available to interested parties, through the European Soil Data Centre (ESDAC) of the Joint Research Centre (JRC), with provision of guidance, datasets and technical support (http://esdac.jrc.ec.europa.eu/themes/g2-model). Up until now, the G2 model has been implemented in five different study areas in SE Europe and in Cyprus. In two of these cases (Crete and Cyprus), pre-existing field data were available either for calibration or rough verifications.

The objective of this paper was to present a complete and detailed description of the G2 erosion model and discuss the experience gained from algorithm development and revisions, and the case-studies conducted since model introduction in 2010. Also, to offer guidance and suggestions to potential users on an appropriate data collection, processing, and analysis. Finally, to examine model's perspectives in Europe and the world after recent improvements in data availability.

## 2 Model overview

G2 is an empirical model for soil erosion rates on month-time intervals and has evolved with time into a quantitative tool with two distinct modules: one for soil loss and one for sediment yield.

The module for soil loss (denoted as G2*los*) inherits its main principles and many of its formulas from the Universal Soil Loss Equation (USLE) (Wischmeier and Smith, 1978) and the Revised-USLE (RUSLE) (Renard et al., 1997). Ferro and Porto (2010) argues that USLE is a robust empirical model with a logical structure regarding the variables used to simulate the physical erosion process. The input datasets of the G2 applications can be derived from geodatabases freely and regularly available by European or other international institutions.

**Fig. 1:**
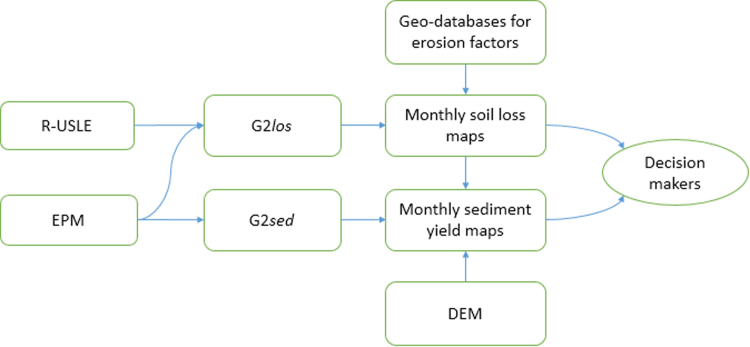
A flowchart of the contribution of R-USLE and EPM models to the modules of G2 and their relation to input and output data.

The module for sediment yield (denoted as G2*sed*) adopts the sediment delivery ratio (SDR) formula from the Erosion Potential Method (EPM) (Gavrilovic, 1988, Marques da Silva et al., 2014). The main input dataset is a high resolution digital elevation model (DEM), from which the required topographic and hydrographic properties can be derived. The G2*sed* module uses the outcome of the G2*los* module and the calculated EPM figures, to produce sediment yield maps (Karydas and Panagos, 2016) (Fig. 1).

G2 model is designed to run in a GIS environment, as it adopts modifications made by Moore and Burch (1986) for spatially distributed soil loss assessments. G2 produces soil loss maps as raster layers at a 100-m resolution and sediment yield maps as vector layers at a 100-ha minimum mapping unit (MMU), on a month-time step.

The spatial scale of application in G2 is affected mainly by the terrain dataset, which has been proved to cause tremendous effect on erosion outputs; see for example, Tantasirin et al. (2016), Barrios and Frances (2012), and Rojas et al. (2008). As therefore, the cell size of the erosion maps is determined by the resolution of the terrain dataset, taking also account of its positional accuracy. For example, a DEM of 25–30 m resolution (e.g. an ASTER-GDEM or a EU-DEM), will allow to map erosion features at a 100-m cell size. Bringing the dataset closely to model specifications, or (inversely) adapting a model to the particularities of the available dataset can be understood as ‘hidden calibration’ of a model, a process inevitable in empirical modelling (Longley et al., 2004).

Temporal scale of G2 is set by default to month-time intervals, instead of yearly assessments originally provided by USLE or EPM; a month is the finest time-step, for which rainfall data could be made available for long periods and wide areas.

## 3 G2*los* module

G2*los* consists of a set of algorithms (adopted, revised, or developed) for producing month-time step maps and statistics of soil loss caused by sheet and interrill erosion processes. Inherited by RUSLE, five input erosion parameters are combined by G2 in a multiplicative equation, to estimate a quantitative erosion output:

(1) $$E_{m}=\frac{R_{m}}{V_{m}}\cdot S\cdot \frac{T}{L}$$

Where E_m_: soil loss for month m (t ha^−1^); R_m_: rainfall erosivity of month m (MJ mm ha^−1^ h^−1^); V_m_: vegetation retention for month m (dimensionless); S: soil erodibility (t ha h MJ^−1^ ha^−1^ mm^−1^); T: terrain influence (dimensionless); L: landscape effect (dimensionless).

Compared to the RUSLE main equation, in Eq. (1):

• R is identical to R of RUSLE;
• V plays a role analogous to that of C in RUSLE (though in an inverse manner, i.e. V~1/C);
• S is identical to K of RUSLE;
• T is identical to LS of RUSLE; and
• L plays a role analogous to that of P in RUSLE (though in an inverse manner, L ~1/P); also, L plays a corrective role to T.

Factors in the numerator of Eq. (1) (i.e., R, S, and T) express natural erosion forces related to the specific site, whereas factors in the denominator (i.e., V and L) express natural or human-induced (related to land management), anti-erosion forces; the product VxL (always ≥ 1) could be seen as a sustainability quantum.

The R and V factors are those with a dynamic character over an annual cycle; R expresses the cumulative erosive effect of all rainfall events in a specific month; whereas, V expresses the protective role of vegetation coverage and proper land use management applied during the same month. The rest factors (T, S, and L) can be considered as static, although Borselli et al. (2012) have reported that soil erodibility shows some seasonal fluctuations. Only R and S are dimensional by default, whereas the rest are dimensionless. Value ranges for R, S (K), and T (LS) are determined by RUSLE equations and nomographs (adapted to the Metric System); while V is always greater than 1 and L ranges between 1 and 2 (Table 1).

**Table 1:** A summary of all mathematical properties of the G2*los* module.

**Factor**	**Role**	**Character**	**Units**	**Range**	**Dimensionality (P: Power; L: Length; M: Mass)**
**R**	Erosive	Dynamic	MJ mm ha^−1^ h^−1^	[0,+∞)	[P][L^−1^]
**V**	Protective	Dynamic	–	[1,+∞)	0
**S**	Erosive	Static	t ha h MJ^−1^ ha^−1^ mm^−1^	(0,0.1)	[M][L^−1^][P^−1^]
**T**	Erosive	Static	–	[0,20]	0
**L**	Protective	Static	–	[1,2]	0
**E**	Erosion	Dynamic	t ha^−1^	[0,+∞)	[M][L^−2^]

## 4 Erosion factors

### 4.1 Rainfall erosivity (R)

According to Wischmeier and Smith (1978), rainfall erosivity (R) is defined as the numerical measure of the erosive potential of rainfalls within a specific period of time. In physical terms, R indicates how particle detachment and transport capacity are combined in the erosive processes. The experimental research conducted by the USLE developers (and also in later times) has proved that soil loss is directly proportional to R, although with moderate correlation (Sonneveld and Nearing, 2003).

Mathematically, the R factor for any given period of time is calculated by summing up the product of total storm energy (E) times the maximum 30-min intensity (I_max30_ or I_30_) of each of the storm events. The formula for storm energy (E) is a logarithmic equation of rainfall intensity (I), which has been extracted from a big set of experimental data (over 10,000 year-plots); this equation has been criticized by Cardei (2010) for dimensionality incompatibilities. Later attempts have resulted in exponential or other non-linear equations of rainfall erosivity (R) vs. rainfall intensity (I) or rainfall volume (P) (Renard and Freimund, 1994; Ferro and Porto, 1999; Diodato and Bellocchi, 1999).

RUSLE has been criticized, also, for not taking adequately into account the volume of rainfall (but only the intensity), thus leading often to overestimations, especially at low rates (Kinnel, 2003). On the other hand, empirical formulas based exclusively on rainfall volume, such as the Arnoldus index (Ferro and Porto, 1999), have been also criticized for being used in different areas from those for which they were developed (Marker et al., 2008).

The introduction of monthly intervals in erosion assessments has been recommended by ‘Agricultural Handbook No 282’ of the USDA, which mentions the potential of seasonal R-distributions over an entire year and suggests that effectiveness in erosion control depends on how ‘the year's erosive rainfall is distributed’ in a specific area (Wischmeier and Smith, 1965).

Because lack of rain intensity data has been a quite common situation, G2 developed initially an alternative methodology for R calculations, by replacing I_30_ in the original R equation of USLE by a simulated monthly rain intensity; the new quantity was defined as the ratio of the total rainfall of a month over the hourly duration of this rainfall in the same month. Earlier, Renard and Freimund (1994) had developed a similar method for monthly R estimations using precipitation datasets in the case of lacking long term rain intensity data.

The hypothesis of a simulated monthly R, was tested in the trans-border Strymonas/Struma case-study (Greece-Bulgaria), with available rain intensity data from a single weather station (Serres, Greece), for eight consecutive years (1980–1987) (unpublished work related to Panagos et al., 2012). The test showed that the simulated R values were close to the true (calculated with the 30’-interval rain intensity data), only for some months (January, May, June, July, August, October, and November), whereas for the rest of months (or for other stations) uncertainty remained unknown. Similar approaches, modified according to the local data availability and particularities, were followed also in another two case-studies conducted in Albania (Karydas et al., 2015a, Karydas et al., 2015b, Zdruli et al., 2016).

As high temporal resolution rainfall data were becoming available, though, the G2 model turned into adopting the R-equations introduced by Brown and Foster (1987), which use a rain intensity parameter, likewise the original USLE equations. Meusburger et al. (2012) have developed scripts for automated computation of the R-factor following Brown and Foster (1987), the mathematical formulas of which, are as follows:

(2) $$R_{m}=\frac{1}{n}\sum_{i=1}^{n}{\left\{\sum_{j=1}^{k}{\left(EI_{30}\right)}_{j}\right\}}_{i}$$

Where R: rainfall erosivity for month m (MJ mm ha^−1^ h^−1^); i: the years recorded; j: the erosive events during month m; and EI_30_ the rainfall erosivity index of event j. The event erosivity (EI_30_) is defined as:

(3) $${\mathrm{EI}}_{30}=\left(\sum_{r=1}^{t}e_{r}v_{r}\right)\cdot I_{30}$$

Where e_r_: rainfall energy per surface unit and per rainfall volume of a predefined time interval (e.g. 10-min) (MJ ha^−1^ mm^−1^); v_r_: the rainfall volume during the predefined time interval (mm); r: predefined time intervals during the rainfall event; and I_30_: the maximum rainfall intensity of the event during a period of 30 min (mm h^−1^). The unit of rainfall energy (e_r_) is calculated for each predefined time interval as follows:

(4) $$e_{r}=0.29\left[1-0.72exp\left(-0.05i_{r}\right)\right]$$

Where i_r_: rainfall intensity during the predefined time interval r (mm h^−1^).

### 4.2 Vegetation retention (V)

The positive effect of natural or agricultural vegetation on soil protection from erosion is associated not only with the presence of vegetation, but also with its management, especially with regard to farming practices. Three types of effects can be identified (Bergsma, 1986): a) the canopy effect, which depends on crown density and closure degree (indicated above the ground), b) the mulch effect of small plants and residues, which functions as a natural carpet (indicated on the ground), and c) the residual effect in terms of remaining organic matter and enhancement of soil structure (indicated below the ground). The latter could explain differences recorded in erosion under various crop rotations.

USLE defines vegetation coverage and management factor (denoted as C-factor) as the ratio of soil loss from land under a specific vegetation management system to the corresponding soil loss under continuous fallow. The C-factor quantifies the effect of vegetation cover and farming practices during a year and originally takes values from empirical tables based on in-situ observations (Wischmeier and Smith, 1978). In the lack of reliable field data over time, however, RUSLE users have developed alternative methodologies, such as use of available land cover/use maps, vegetation mapping using image classification techniques, or use of various vegetation indices (Vrieling, 2006). These methodologies have been criticized for producing static outputs and for being based on incompatible sources (Panagos et al., 2012), or for resulting in poor correlations with vegetation attributes, especially when applied in Mediterranean environments (Vrieling, 2006).

Introducing an algorithm for a realistic estimation of vegetation's retention effect, was one of the main foci of G2. Set in the denominator of the main G2 formula (Eq. (1)), V-factor (after ‘vegetation’) is by default inversely analogous to C-factor of RUSLE. Mathematically, V-factor is a function of quantified parameters for vegetation and land use.

Initially, a combination of FCover and Leaf Area Index (LAI) was introduced to express vegetation extent and density, respectively. FCover is defined as the fraction of the background covered by green vegetation of the overstorey and understorey as seen from the nadir to the surface (Gutman and Ignatov, 1998). Practically, FCover quantifies the spatial extent of vegetation in a normalized manner. Panagos et al. (2015a) have argued that use of a proxy vegetation layer allows for the quantification of the impact of vegetation cover and management. G2 has adopted the use of month-time step FCover layers to express seasonal changes of vegetation cover. LAI is defined as the one-sided green leaf area per unit ground surface area in broadleaf canopies (Watson, 1947).

However, the two layers (FCover and LAI) demonstrated high spatial and temporal dependency and therefore, LAI was removed from the V formula of G2 after the first case-study. Moreover, Wilhelm et al. (2000) report systematic LAI underestimation when very dense canopies are captured by satellite imagery.

As a replacement of LAI, a new parameter was introduced, namely LU (after ‘land use’), in order to broaden the estimation of effect from vegetation to land use management. In this direction, G2 has elaborated a matrix of indicative LU-values for the most common land uses, based on an interpretation of the EPM empirical datasets (Gavrilovic, 1988) in terms of the CORINE Land Cover nomenclature; this matrix is being enriched empirically over time (Panagos et al., 2015a). Considering that V is set in the denominator of the main equation (whereas, in EPM the conservation coefficient X or Xa, is in the numerator), LU was quantified using a linear inversion of X (or Xa) from a [0−1] into a [1,10] scale, according to the following equation (indicative examples in Table 2):

(5) $$\begin{array}{c} LU=-10*X+11,\;if\;X\geq 0.1\\ LU=10,\;if\;X<0.1 \end{array}$$

**Table 2:** Indicative examples of the conversion of EPM conservation coefficients into LU values.

**EPM land use descriptions**	**Conservation coefficient**		**Corresponding CORINE LC codes**	**LU**
	**Without measures (X)**	**With measures (Xa)**		
Degraded woods under bush with eroded soil	0.60	–	322/324	5.0
Mountain pastures	0.60	–	321	5.0
Good woods on slopes	0.20	–	311/312/313	9.0
Contour farming with mulching	–	0.540	211/212	5.6
Contour orchards	–	0.315	222/223	7.8
Vineyards	(0.70)	0.315	221	(4.0) 7.8
Grazing, meadow amelioration	–	0.300	231	8.0

Furthermore, G2 has introduced an extra land cover parameter, namely degree of imperviousness (in per cent), in order to improve mapping precision within mixed pixels of artificial surfaces with vegetation patches. Provided that artificial surfaces are by default non-erosive surfaces, use of only CORINE LC would exclude these surfaces from mapping. This artefact can be avoided by the combined function of two characteristics that the extra parameter has, i.e.:

• The higher resolution of the imperviousness layers (20 m) compared to the current rough-scale CORINE LC layers (250 m), which allows for downscaling V values significantly.
• The broader numerical range of the imperviousness values (0−100) compared to the narrow range of LU (1−10), which allows for increasing precision of V values.

According to the above developments and considerations, the equation of V has been formulated as follows:

(6) $$V_{mj}={\left(1-imp\right)}^{-1}\cdot e^{\left(LU_{j}\cdot FC_{m}\right)}$$

Where V_mj_: V-factor for month m and land cover/use j, in range [1,+∞) (dimensionless); imp: imperviousness degree corresponding to 0–100% of soil sealing, in range [0,1) (dimensionless); LU_j_: empirical parameter for land use j, in range [1,10], with lower values corresponding to intensive management or unprotected land uses and higher values corresponding to better management conditions; and FC_m_: Fractional vegetation cover for month m, in range [0,1] (dimensionless).

**Fig. 2:**
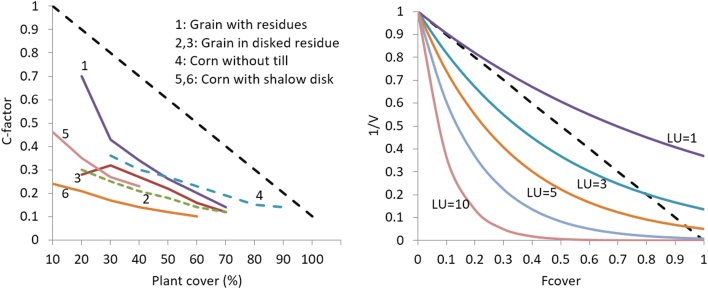
Extraction of exponential equation family of V-factor vs. Fcover for different LU values by G2 (right), according to C-factor values derived from USLE experimental data for indicative crop management practices (left).

The exponential shape of Eq. (6) is imposed by the relation revealed when C-factor values are plotted against plant cover experimental data of USLE (Wischmeier and Smith, 1978). This relation seems to follow a non-linear shape along the entire range of possible values at both axis, while different management practices approximate a family of equations (Fig. 2). Elwell (1978) has used exponential equations to estimate C-factor values for grasslands and pastures, while Zurina et al. (2015) has showed a logarithmic character of the equation linking C-factor with vegetation cover using aerial photographs. In contrast, De Asis and Omasa (2007) have estimated C-factor as a linear function of the fractional abundance of bare soil and ground cover using Landsat imagery.

Manipulation of the LU parameter can account for possible differentiations in crop management (such as use of mulch), provided that this kind of information is known to the model users. Other differentiations (such as grass maintenance after harvest) will be captured by FCover layers of the specific months. Therefore, it is up to the user to calibrate G2 in respect to the very local conditions.

### 4.3 Soil erodibility (S)

As defined by USLE, soil erodibility factor (S, denoted as K by USLE) is the rate of soil loss per unit of R, as measured on a unit plot under specific conditions (the so called ‘Wischmeier plot’) (Wischmeier and Smith, 1978). For soil erodibility estimations, RUSLE provides the following empirical equation (here adapted to the Metric system, SI), which is resolved also by nomographs (Renard et al., 1997):

(7) $$S=0.1317*\left\{\left[2.1*10^{-4}*M^{1.14}*\left(12–OM\right)+3.25*\left(s-2\right)+2.5*\left(p-3\right)\right]/100\right\}$$

Where, K: soil erodibility (t ha h ha^−1^ MJ^−1^ mm^−1^); M: textural factor defined as percentage of silt plus very fine sand fraction content (0.002–0.1 mm) multiplied by the factor: 100 - clay fraction; OM: organic matter content in per cent (%); s: soil structure class (s = 1: very fine granular, s = 2: fine granular, s = 3: medium or coarse granular, s = 4: blocky, platy or massive); and p permeability class (p = 1: very rapid, …, p = 6: very slow).

The availability of geospatial layers for all required input soil physical properties by ESDAC (Panagos et al., 2014a) allows the direct estimation of soil erodibility. Prior to this possibility, G2 applied empirical methods for estimating soil erodibility, based on converting textural classes into S-factor values (Van der Knijff et al., 1999) and then incorporating crusting (Le Bissonnais et al., 1998) and organic matter content (Panagos et al., 2012).

### 4.4 Terrain influence (T)

As a gravity-triggered process, erosion is influenced by terrain according to the degree, the length, and the site-specific shape of the slope. G2 has adopted the Desmet and Grovers (1996) formula for a quantitative estimation of terrain influence (T, denoted as LS by RUSLE):

(8) $$T={\left(\frac{A_{S}}{22.13}\right)}^{0.4}*{\left(\frac{\sin\;b}{0.0896}\right)}^{1.3}$$

Where, T: terrain influence (dimensionless, ≥ 0); A_s_: unit contributing area (or flow accumulation), defined as the surface upstream flowing into a specific unit surface (dimensionless, > 0); and b: slope gradient at the unit surface (rad).

This formula is an adaptation of the Moore and Burch (1986) method for spatially distributed USLE applications, to grid datasets. Using a digital elevation model (DEM) as a grid dataset, the method is considered to estimate T values equivalent to those taken from the original USLE formulas.

However, accuracy errors inherent in DEMs will be propagated to A_s_ and b, then to T, and finally to erosion outputs (Abd Aziz et al., 2012). In order to compensate for these errors, G2 suggests calibration of T values according to USLE experimental results for irregular slopes (i.e. convex or concave terrain continuums); for example, a concave terrain continuum up to 15% of slope gradient, is expected to give a 3.5 value for T (Wischmeier and Smith, 1978). Furthermore, values greater than 20 (a threshold implied from the LS-nomograph of USLE), are considered to be identical either to concentrated water flow, very steep slopes, or geologic discontinuations and should be masked out from the T-layer.

According to a review conducted by Karydas et al. (2014), the adoption of a unit contributing area (or flow accumulation) method classifies G2 in the ‘pathway models’, i.e. the models respecting a well-defined transport process between sources and receptors.

### 4.5 Landscape effect (L)

G2 has introduced the L-factor (after ‘landscape’), which accounts for the effect of land cover/use alterations on erosion (Panagos et al., 2012). Landscape effect (L) quantifies the possibility of linear landscape features to interrupt rainfall runoff due to their function as obstacles to water motion. The importance of this property for erosion has been recognised also by Bergsma (1986), who states: “Many times, field boundaries modify slope length, thus interrupting the overland flow”.

In this sense, L-factor can be considered as analogous (in an inverse manner) to the support practice factor (P) of RUSLE, which quantifies conservation measures taken by land managers; and also, as corrective to the terrain influence (T), specifically for slope length, which may be reduced due to landscape alterations. ‘Landscape effect’ replaces the term ‘Slope intercept’ (denoted by I), which corresponds exactly to the same quantity. Replacement was found necessary, though, considering that the term ‘Slope intercept’ emphasised more the corrective role of the L-factor against T, rather than its main role, which is to quantify landscape functions (and is analogous to P).

L-layer is calculated using a 3 × 3 Sofel filter (a non-directional edge-detection filter) applied on the Near-Infrared (NIR) band of a satellite image of similar or higher resolution to that of the DEM in use. Landscape features potentially intercepting slope and –at the same time- capable to be captured by a spatial filter may include paved roads, paths and strips between fields, fences, natural hedges, terraces, etc. The effect of possible landscape alterations is quantified by the ratio of the calculated filter value over the theoretic maximum value of the image. According to the above approach, the following formula was found appropriate for computing L-layer:

(9) $$L=1+\sqrt{S_{f}/DN_{\max}}$$

**Fig. 3:**
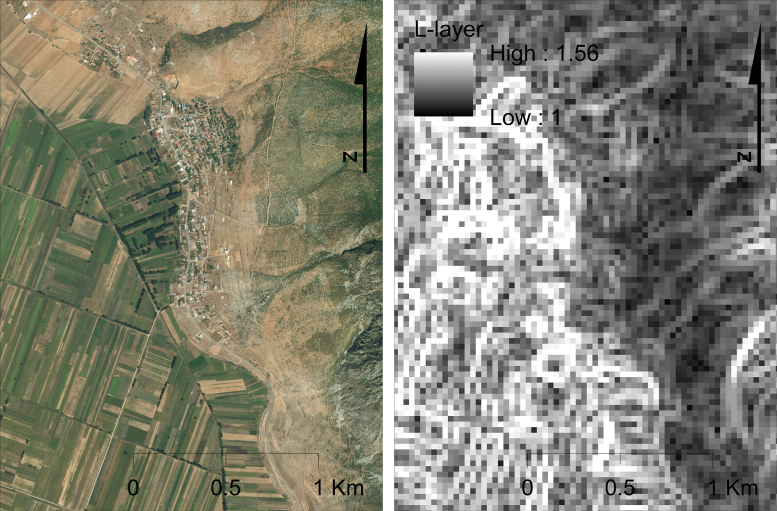
The protective effect of the landscape alterations on erosion in a subset of a study area in Albania); left: high resolution basemap; right: L-layer derived from Landsat-8 imagery.

Where, L: landscape effect in range [1,2] (dimensionless); S_f_: Sobel filter value in range [0, DN_max_] (dimensionless); and DN_max_: theoretic maximum digital number of the image (e.g. 255 for 8-bit recording systems) (dimensionless) (Fig. 3).

According to Eq. (9), the L-values range in [1,2], which may be translated as a possible reduction of soil loss due to landscape alterations up to the half of the original values. This can be justified by the fact that landscape alterations may affect erosion output only partially (in terms of ‘between-pixels’ erosion) and therefore, their effect is expected to be low to moderate, fitting with the complementary and corrective role of L-factor in the main G2 equation. The application of a root square to the ratio, results in an exaggeration of the values, particularly the lower ones. Indicatively, the mean value of L-factor in the case-study of Crete, a highly heterogeneous environment, was estimated equal to 1.12, resulting in an erosion reduction by 11% on average (Panagos et al., 2014b).

## 5 G2*sed* module

The G2*sed* module is designed to map area-specific sediment yield (SSY) (or simply, sediment yield rates) at a watershed scale on month-time intervals. While sediment yield (SY) measures an absolute amount of sediments produced within a watershed, SSY is defined as SY per areal unit (De Vente and Poesen, 2005, De Vente et al., 2008). SSY layers are calculated by G2 according to the following equation (Borrelli et al., 2014):

(10) $$SSY_{m}=SDR\cdot E_{m}$$

Where, SSY_m_: area-specific sediment yield (sediment yield rate) during month m (t ha^−1^); SDR: sediment delivery ratio, in range [0,1] (values differentiated per watershed, dimensionless); E_m_: soil loss rate for month m (t ha^−1^).

For calculating SDR of a watershed, G2*sed* has adopted the Erosion Potential Method (EPM) (Gavrilovic, 1988). Experimental validation of EPM in Swiss Alps, conducted by De Cesare et al. (1998), has resulted in R^2^=0.86 explanation of variance. Τhe main equation of EPM for SDR of a watershed, is as follows (De Vente and Poesen, 2005):

(11) $$SDR=\frac{\sqrt{P\cdot Z}\cdot (L_{p}+L_{s})}{(L_{p}+10)\cdot A}$$

Where, SDR: sediment delivery ratio, in range [0,1] (dimensionless); P: perimeter (km); Z: difference of minimum from mean altitude (km); L_p_: total length of the primary stream segments (km); L_s_: total length of the secondary stream segments (km); and A: projective surface (km^2^).

In order to derive all required hydrographic and topographic input parameters for the SDR equation, a digital elevation model (DEM) can be employed. For extracting watersheds and stream network from a DEM, G2 applies the D8 algorithm (Jenson and Domingue, 1988), which according to Ariza-Villaverde et al. (2015), results in realistic features of flow conditions and consistent flow patterns.

The scale at which hydrographic features will be extracted from a DEM, though, depends on its resolution and can be regulated by an empirical threshold set to flow accumulation layer (which is an intermediate derivative of the process). According to a rule of thumb suggested by Dobos and Daroussin (2005) the average watershed surface may be about 100 times the surface of the DEM's cell.

However, EPM has been developed for river basins, which are found usually at different scale from the scale of watersheds extracted according to the rule of Dobos and Daroussin (2005). Actually, the application of G2*sed* in Cyprus showed that SDR was affected strongly by the size of the watersheds: for a mean watershed size of 0.625 km^2^ (corresponding to the finest possible scale for the given DEM of 25 m resolution), the mean SDR value was found to be 0.10; whereas, for a mean watershed size of 12.5 km^2^ (corresponding to the river basin scale), the mean SDR was 0.26 (Karydas and Panagos, 2016).

**Fig. 4:**
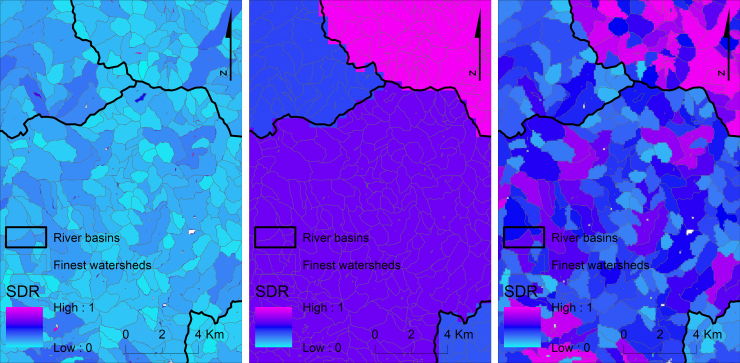
Calculation of SDR per watershed using G2*sed*; from left to right: original SDR values at watershed scale; SDR values calculated per river basin; adjusted SDR values at watershed scale according to the containing river basin (subset from the application in Cyprus).

In order to compromise method appropriateness with the required spatial detail, G2 recommends calculation of SDR at two different scales: the river basin scale and the finest possible watershed scale. Then, SDR values at the finest watershed scale can be adjusted arithmetically, by multiplying with the ratio of the two SDRs (SDR of the river basin in which the watersheds are contained over SDR of every specific watershed in the same basin). In summary, G2 takes the river basins as a baseline scale and then downscales the results to the small watersheds (Fig. 4).

Indication of stream network order in G2*sed* is achieved by applying the Strahler method (Strahler, 1952). According to this method, when two segments of the same order joint, they form a segment of the next-lower order (e.g. when two 3rd order segments joint, they form a 2nd order segment); when two streams of different order joint, they form a stream of the lowest order between the two joint segments; every network segment reaching the watershed outlet is considered to be of 1st order. For capturing an adequately detailed 2nd order drainage network (required for Eq. (11)), the threshold of flow accumulation should be set slightly lower than that set for watershed extraction (e.g. 300 cells if 500 is set for watersheds).

Integrating G2*sed* equations for sediment transfer and a known risk index for heavy metals (Hakanson, 1980), a new algorithm for estimating risk by heavy metals, was developed by Karydas et al., 2015a, Karydas et al., 2015b. The new method, namely G2*met*, can be considered as a third module of G2, as it deploys the entire set of G2 algorithms to provide monthly risk maps for heavy metals.

## 6 Available input data

Data requirements of G2 are becoming more and more possible to be met, as long as appropriate geodatabases are made available to decision makers by European or other international institutions. Indicative examples of free, regularly updated, validated, and ready-to-use geospatial datasets, for the preparation of the erosion factor layers for G2, include:

• FCover from BIOPAR layers available by the Copernicus Land Monitoring Service (CLMS); BIOPAR layers are created with the SAIL/PROSPECT baseline vegetation model (Verhoef, 1985).
• CORINE Land Cover (CLC) available by CLMS;
• Imperviousness layer from the High Resolution Layers (HRL) available by CLMS;
• Land Use/Cover Area frame Survey (LUCAS) (the soil component) available by ESDAC;
• ASTER-GDEM and EU-DEM (elevation raster data) available by METI/NASA and Eurostat, respectively; and
• Sentinel-2 (S2) satellite imagery available by the European Space Agency (ESA).

Furthermore, JRC has developed GIS-ready layers of rainfall erosivity on month-time intervals, at different resolutions for the European Union (500 m) and the Globe (1 km), namely the Rainfall Erosivity Database at European Scale (REDES) and the Global Rainfall Erosivity Database (GloREDa), respectively, available by the European Soil Data Centre (ESDAC) (Ballabio et al., 2017, Panagos et al., 2017). JRC has also developed the soil erodibility layer at a 500-m resolution (Panagos et al., 2014a) and the terrain influence layer on a 25-m resolution (Panagos et al., 2015b) covering the European Union; both datasets are available in ESDAC (Table 3).

**Table 3:** Input parameters, processes, and data sources for the preparation of erosion factors of G2 (GIS-ready layers in *italics*).

**Erosion factor**	**Input parameters**	**Processes**	**Data source**	**Cell/Pixel/MMU**	**Time step updates**	**URL**
R-factor	v_r_	R calculation at meteo-stations, Interpolation with rainfall surfaces	EU: *REDES* (ESDAC)	500 m (Europe)	5–60 min	http://esdac.jrc.ec.europa.eu/content/rainfall-erosivity-european-union-and-switzerland
	i_r_		Globe: *GLoREDa* (ESDAC)	1 km (Global)		http://esdac.jrc.ec.europa.eu/content/global-rainfall-erosivity
V-factor	FC	Normalized value [0,1], > 4-year sequence	EU: BIOPAR/PROBA-V (CLMS)	PROBA-V: 333 m, SPOT-VGT: > 1 km, Image: < 30 m	10 days	http://land.copernicus.eu/global/products
			Globe: BIOPAR/SPOT-VGT (CLMS)			
			Sentinel-2 (ESA/CLMS)	10 m		https://scihub.copernicus.eu/dhus/#/home
	LU	Conversion of LC class to LU value (matrix)	EU: CLC (CLMS)	250 m/25 ha	EU: 6 years	http://land.copernicus.eu/pan-european/corine-land-cover
	imp	Normalized value [0,1]	EU: HRL (CLMS)	20 m	EU: 3 years	http://land.copernicus.eu/pan-european/high-resolution-layers/imperviousness/view
S-factor	M	Conversion of texture class to M value (matrix),	EU: Soil properties (ESDAC)	500 m	EU: Reference year: 2009	http://esdac.jrc.ec.europa.eu/content/european-soil-database-v20-vector-and-attribute-data
	OM	Effect of crust and organic matter	EU: *Soil erodibility* (LUCAS/ESDAC)			http://esdac.jrc.ec.europa.eu/themes/soil-erodibility-europe
T-factor	A_s_	D8 method, slope as %	ASTER GDEM (METI-NASA), EU-DEM (Eurostat)	30 m, 25 m	Reference years: 2009,	https://asterweb.jpl.nasa.gov/gdem.asp
	b		EU: *Terrain factor* (ESDAC)	25 m	2014	http://esdac.jrc.ec.europa.eu/content/ls-factor-slope-length-and-steepness-factor-eu
L-factor	S_b_	3×3 Sobel filtering	Sentinel-2 (ESA/CLMS)	10 m in NIR	5–10 days	https://scihub.copernicus.eu/dhus/#/home

Among the erosion factor layers, quality of R and S depend strongly on original point data, irrespectively whether or not the interpolated geospatial layers were already available (JRC) or were created by the model users.

As far as V-surfaces are concerned, use of FCover layers from different satellites (such as MERIS, PROBA-V, and SPOT-VGT) and for many consecutive years (five or more) is highly recommended. Variance in FCover sources allows to eliminate gaps from cloudiness and also to compensate for inter-annual discrepancies (usually detected in agricultural lands).

In the same direction, the contribution of a high resolution FCover layer is also encouraged. Apart from filling the gaps, high resolution FCover layers could be used to effectively downscale the final FCover outputs. Sentinel-2 imagery could be considered as an appropriate solution in this direction. Calculation of FCover values from this image type can be realised using empirical equations (e.g. Verger et al., 2015).

The LU values can be taken from a matrix developed by G2, where the most common CORINE categories at Level 3 are interpreted in terms of a degree of land use influence on erosion. Recently, this matrix was revised in accordance with a new C-factor dataset elaborated by the JRC for the entire European Union (Panagos et al., 2015a). However, for several land uses, the values of the two sources remain different (Table 4). The selected LU values will be assigned to the corresponding CORINE or other equivalent layer polygons.

**Table 4:** A matrix of the proposed LU values by G2 for the most common CORINE classes and the corresponding C-factor values proposed by the JRC database (equivalent to converted LU values).

**CORINE level 3**	**CORINE code**	**Mean LU proposed by G2**	**LU converted into C**^a^	**Mean C proposed by JRC**
*Continuous urban fabric*^b^	*111*	*10*	*0.10*	–
*Discontinuous urban fabric*	*112*	*10*	*0.10*	–
*Industrial or commercial units*	*121*	*10*	*0.10*	–
*Road and rail networks and associated land*	*122*	*10*	*0.10*	–
*Port areas*	*123*	*10*	*0.10*	–
*Airports*	*124*	*10*	*0.10*	–
*Mineral extraction sites*	*131*	*1*	*1.00*	–
*Dump sites*	*132*	*1*	*1.00*	–
*Construction sites*	*133*	*1*	*1.00*	–
*Green urban areas*	*141*	*10*	*0.10*	–
*Sport and leisure facilities*	*142*	*10*	*0.10*	–
Non-irrigated arable land	211	5.5	0.18	0.20
Permanently irrigated land	212	1	1.00	0.28
Rice fields	213	1	1.00	0.15
Vineyards	221	3.5	0.28	0.35
Fruit trees and berry plantations	222	4.5	0.22	0.22
Olive groves	223	4.5	0.22	0.23
Pastures	231	9.5	0.11	0.09
Annual crops associated with permanent crops	241	5.5	0.18	0.23
Complex cultivation patterns	242	7.0	0.14	0.14
Agricultural land with natural vegetation	243	6.5	0.15	0.12
Broad-leaved forest	311	10	0.10	0.001
Coniferous forest	312	10	0.10	0.001
Mixed forest	313	10	0.10	0.001
Natural grasslands	321	8	0.12	0.04
Moors and heathland	322	7	0.14	0.04
Sclerophyllous vegetation	323	9	0.11	0.06
Transitional woodland-shrub	324	7	0.14	0.02
Beaches, dunes, sands	331	5	0.20	–
*Bare rocks*	*332*	*7*	*0.14*	–
Sparsely vegetated areas	333	7	0.14	0.26
Burnt areas	334	7	0.14	0.34
*Inland marshes*	*411*	*1*	*1.00*	–
*Salt marshes*	*421*	*1*	*1.00*	–
*Salines*	*422*	*1*	*1.00*	–
*Water courses*	*511*	*1*	*1.00*	–
*Water bodies*	*512*	*1*	*1.00*	–
*Coastal lagoons*	*521*	*1*	*1.00*	–
*Estuaries*	*522*	*1*	*1.00*	–
*Sea and ocean*	*523*	*1*	*1.00*	–

a conversion is possible only under the condition of FCover = 1.

b in italics, by default non-erosive land cover/uses; treated as erosive.

G2 treats all the CORINE categories originally as erosive, although some of them are considered to be non-erosive, e.g. artificial surfaces or water bodies. This is due to the possibility of mixed pixels in the available raster data, i.e. the existence of heterogeneous surfaces contained in a single pixel (usually found at the boundaries of the categories). Taking, therefore, all the categories as erosive by default, the risk of neglecting an erosive surface (contained in a mixed pixel) is avoided; the V value of this surface will be judged by the rest contributing parameters, i.e. fraction of vegetation cover (FCover) and imperviousness degree (imp).

**Fig. 5:**
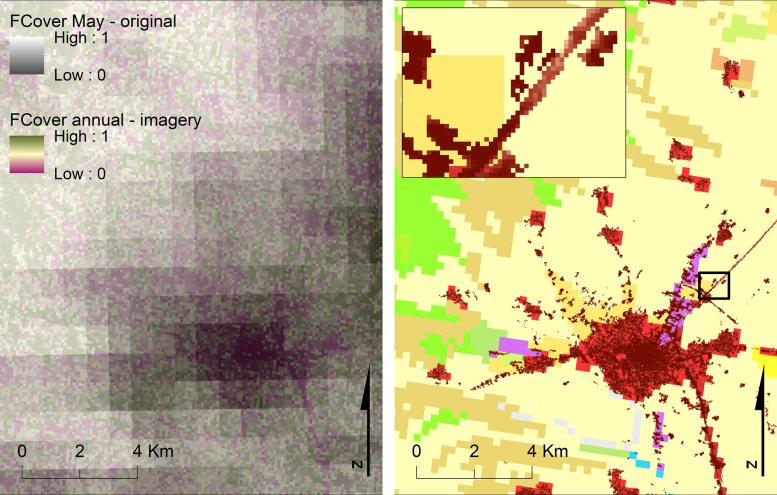
Overlay of geospatial layers contributing to V-factor; left: FCover derived from SPOT-VGT (890 m) overlaid on FCover derived from high resolution satellite imagery (then resampled to 100 m); right: Imperviousness HRL (20 m) overlaid on CORINE LC 2012 (raster, at 250 m) (study area in Greece).

The high resolution imperviousness-degree layers can be derived from Copernicus Land Monitoring Service, as HRLs (at a 20-m cell size). These layers indicate the degree of sealed soil within every pixel in a range [0−1] (corresponding to 0–100% of soil sealing). It is suggested to mask out cells with a value of 0 (if any) or convert them to no-data in order to ensure the arithmetical integrity of the V-function (possible null imperviousness values would render zero V and thus the denominator). Complementarity of the geospatial layers contributing to V-surfaces (FCover, LU, and imp) is considered to improve overall accuracy of V-factor (Fig. 5).

For S-factor, Texture, Organic Matter, Soil Structure, and Permeability layers can be derived from the European Soil Data Centre (ESDAC) of the JRC for the EU at 500-m resolution grids. If extra local soil datasets are available, these can be used to correct, refine, or downscale the original rough S estimations. In case of lack of the above data, the method introduced by Van der Knijff et al. (1999) and improved by Le Bissonnais et al. (1998) and Panagos et al. (2012) could be applied as an alternative.

For T-factor and the SDR estimations (G2*sed* module), use of ASTER-GDEM or EU-DEM, both validated and with a spatial resolution better than 30 m, is recommended.

For L-factor, a recent satellite image of medium-high resolution, such as Sentinel-2, is considered appropriate to capture up-to-date landscape alterations. Use of the near-infrared (NIR) band of the image is recommended, as this band is sensitive both to vegetation and water variations. Furthermore, use of images from different dates –if available- and extraction of an averaged L is welcomed. On the contrary, extraction of a principal component of the image bands is not recommended.

For optimum consistency of the input geospatial layers, the UTM WGS84 projection system is suggested. Also, a buffer zone of 1 km from the coastline (or other known discontinuities) should be masked out from the final erosion outputs. This would help to avoid biases implied from spatial interpolation or extrapolation methodologies implemented on R or S point data or possible existence of tiny coastline watersheds.

## 7 G2 case-studies

**Fig. 6:**
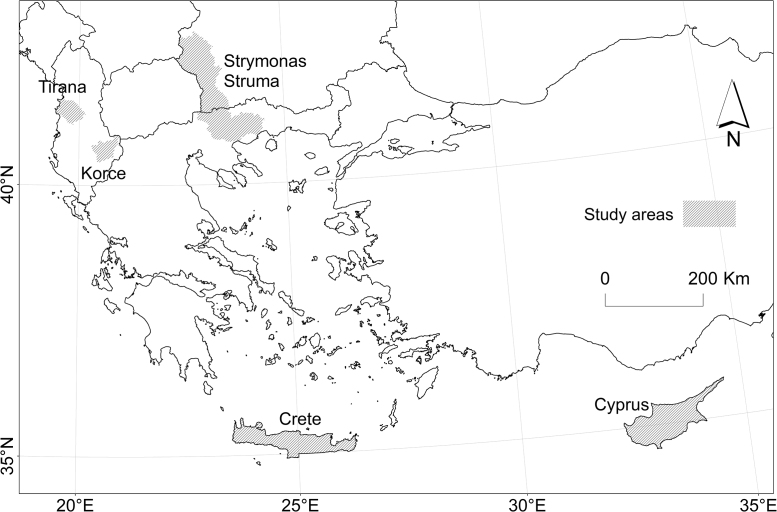
The study areas of the G2 model applications.

The G2 model has been developed and revised through five published case-studies conducted in the South East Europe and Cyprus (Fig. 6), with a steady view to serve as an effective decision-making tool based on harmonized datasets and procedures (Table 5).

**Table 5:** The case-studies carried out with the G2 erosion model and indicative input and output parameters.

**Study area**	**Extent (km**^**2**^**)**	**R-surfaces**	**V-surfaces**	**S-surfaces**	**T-surfaces**	**L-surfaces**	**Erosion map resolution (m)**	**Annual soil loss (t ha**^**−1**^**)**	**Watershed size (km**^**2**^**)**	**Annual SSY (t ha**^**−1**^**)**
Strymonas/Struma river basin	14,500	1 station per 580 km^2^	FSoil: 300 m, LAI: 300 m (MERIS)	1 sample per 278 km^2^ (ESDB, National data)	ASTER GDEM (30 m)	Image 2006 mosaic (25 m)	300	8.75	–	–
Crete island	8336	1 station per 347 km^2^	FCover: 300 m (MERIS)	1 sample per 91 km^2^ (LUCAS Topsoil)	ASTER GDEM (30 m)	Image 2006 mosaic (25 m)	300	8.12	–	–
		WorldClim covariates	CORINE2006 (25 ha)							
Ishmi-Erzeni river basin	2200	1 station per 190 km^2^	FCover: 3.5 km (SPOT-VGT)	1 sample per 46 km^2^	Local topographic maps (250 m contour)	Landsat 8 (30 m)	300	6.50	–	–
			Landsat 7 classification							
Cyprus	9251	1 station per 264 km^2^	FCover: 3.5 km (SPOT-VGT	1 sample per 102 km^2^ (LUCAS Topsoil)	ASTER GDEM (30 m)	Landsat 8 (30 m)	100	11.75	0.625	3.32
		WorldClim covariates	CORINE2006 (25 ha)							
Korce region	1690	1 station per 338 km^2^	FCover: 3.5 km (SPOT-VGT)	1 sample per 6.5 km^2^	ASTER GDEM (30 m)	Landsat 8 (30 m)	30	10.25	–	–
		WorldClim	CORINE2006 (25 ha)							

The main challenge of the first case-study, carried out in the cross-border Strymonas/Struma river basin (Greece-Bulgaria) was data harmonization from different countries. The model was focused on developing an alternative formula for R-factor based on the introduction of a simulated monthly R parameter in the RUSLE R-equation. Also, it deployed FSoil (fraction of bare soil, algebraically complementary to FCover) as the main vegetation dataset and LAI as the secondary one, combined in a non-linear function, for the calculation of month-step V-surfaces.

In the second case-study (Crete, Greece), the main erosion equation was revised into the current shape, with V and L (former I) moving to the denominator (Panagos et al., 2014b). In this study, two major developments took place: a) the simulated R methodology was replaced by the well-established R-methodology of Brown and Foster (1987), computerized by Meusburger et al. (2012); and b) V-factor was improved after introduction of the LU parameter (in replacement of LAI) and the modification of V-equation into an exponential function in accordance to the USLE experimental database. The latter facilitated detection of erosion hot-spots associated with specific land uses (e.g. intensification of grazing in natural grasslands).

In the third case-study, conducted in Ishmi-Erzeni river basin (Tirana, Albania), the main focus was put on detecting erosion hot-spots caused by rapid land use changes, especially the extreme urbanization in low areas. However, the only available vegetation dataset on a monthly basis were FCover layers from SPOT-VGT at a 3.5 km cell size. In order to compensate for such a rough resolution, recent Landsat imagery was used for an up to date, medium-high resolution land cover/use classification, thus downscaling the originally rough FCover layers to the targeted scale of 300 m (Karydas et al., 2015a, Karydas et al., 2015b).

The fourth case study (Cyprus) was the first country-scale application of the G2 model. As Cyprus is an island, a complete watershed analysis was possible and therefore ideal to develop a module for sediment yield assessments on month-time intervals, namely the G2*sed*. EPM was employed for a second time by G2 (the first one was for the formulation of LU matrix). Preparatory work for the G2*sed* module was done by Karydas et al., 2015a, Karydas et al., 2015b, towards the development of a new algorithm for risk mapping of heavy metals (namely, the G2*met* module). In Cyprus, the erosion maps produced by G2*los* were extracted for first time at 100 m (from 300 m before). This was found necessary, in order to match with the small mean watershed size (0.625 km^2^ on average) targeted by the application (Karydas and Panagos, 2016).

In the fifth case-study (Korce, Albania), the very high density of the available soil data (one soil sample per 6.5 km^2^) allowed for first time, downscaling the output maps to a 30-m resolution. Beyond that, the study emphasised extraction of detailed erosion statistics per land cover/use according to the CORINE nomenclature (Zdruli et al., 2016).

In most cases, adequate field data were not available for a comprehensive accuracy test of the G2 applications. Only in two cases (in Crete and Cyprus), limited pre-existing field data were available, either for model calibration or rough verifications of the outputs. Pre-existing field data, however, are not always appropriate for realistic validation tests of big-scale erosion applications; in many cases, they are also outdated.

## 8 Conclusions

The G2 model has adopted fundamental equations on rainfall erosivity (R), soil erodibility (S), and terrain influence (T) from RUSLE; and sediment delivery ratio (SDR) from EPM. In parallel, it has introduced a new formula for vegetation retention (V, inversely analogous to the vegetation cover and management factor, C) based on empirical data from USLE. Finally, the support practice factor (P) of USLE has been replaced by a new factor, namely the landscape effect (L); the latter is also corrective to the terrain influence. All input parameters can be derived from geodatabases regularly updated and available by European or other international institutions (mainly through CLMS and ESDAC).

Recent development of GIS-ready layers for rainfall erosivity on month-time intervals (Panagos et al., 2015c, Ballabio et al., 2017), soil erodibility (Panagos et al., 2014a) and terrain influence (Panagos, 2015b) by the JRC, on a European Union or global scale (Panagos et al., 2017) is expected to facilitate G2 applications significantly. On the other hand, JRC's annual C-factor maps (Panagos et al., 2015a) cannot be considered as appropriate for G2 applications, for which seasonality is a prerequisite.

For vegetation retention (V), G2 has elaborated its own sophisticated approach by combining three different sub-factors (fractional vegetation cover, land use, and imperviousness degree), instead of using a single predefined value per land cover/use or management practice. In favour of emphasising V-factor estimation is the remark of Garcia-Ruiz (2010) that “among the factors explaining the intensity of soil erosion, plant cover and land uses are considered the most important, exceeding the influence of rainfall intensity and slope gradient”. Moreover, Estrada-Carmona et al. (2017) argue that C-factor produces the greatest degree of variation in RUSLE-based predictions and this factor can be improved with the application of better land management practices and agro-environmental friendly measures (Borrelli et al., 2016). It is expected that broad availability of high resolution Sentinel-2 multispectral imagery by ESA, will soon allow replacement of the currently used low resolution FCover layers. The same data could be deployed to produce multiple L-factor layers, possibly at month-time step, likewise the R and V factors.

G2 flexibility in spatial scale (from few square kilometres to a country) combined with the provision of month-time step assessments, is expected to improve understanding of erosion processes, especially in relation to land uses and climate change. In this view, G2 could contribute to large-scale soil degradation assessments, such as the one introduced by Sarapatka and Bednar (2015) for Czech Republic.

The G2 model can be considered as a complete, quantified, integrated algorithm for soil loss and sediment yield assessments on a month-time step, which has full potential to support decision-makers with standardised maps on a regular basis. The current document can be used as a baseline guidance to model users, who could be supported further by the JRC/ESDAC.
